# Distinct actions of ancestral vinclozolin and juvenile stress on neural gene expression in the male rat

**DOI:** 10.3389/fgene.2015.00056

**Published:** 2015-03-02

**Authors:** Ross Gillette, Isaac Miller-Crews, Michael K. Skinner, David Crews

**Affiliations:** ^1^Institute for Cellular and Molecular Biology, The University of Texas at AustinAustin, TX, USA; ^2^Center for Reproductive Biology, School of Biological Sciences, Washington State UniversityPullman, WA, USA; ^3^Department of Integrative Biology, The University of Texas at AustinAustin, TX, USA

**Keywords:** endocrine disruption, amygdala, hypothalamus, transgenerational, vinclozolin

## Abstract

Exposure to the endocrine disrupting chemical vinclozolin during gestation of an F0 generation and/or chronic restraint stress during adolescence of the F3 descendants affects behavior, physiology, and gene expression in the brain. Genes related to the networks of growth factors, signaling peptides, and receptors, steroid hormone receptors and enzymes, and epigenetic related factors were measured using quantitative polymerase chain reaction via Taqman low density arrays targeting 48 genes in the central amygdaloid nucleus, medial amygdaloid nucleus, medial preoptic area (mPOA), lateral hypothalamus (LH), and the ventromedial nucleus of the hypothalamus. We found that growth factors are particularly vulnerable to ancestral exposure in the central and medial amygdala; restraint stress during adolescence affected neural growth factors in the medial amygdala. Signaling peptides were affected by both ancestral exposure and stress during adolescence primarily in hypothalamic nuclei. Steroid hormone receptors and enzymes were strongly affected by restraint stress in the mPOA. Epigenetic related genes were affected by stress in the ventromedial nucleus and by both ancestral exposure and stress during adolescence independently in the central amygdala. It is noteworthy that the LH showed no effects of either manipulation. Gene expression is discussed in the context of behavioral and physiological measures previously published.

## INTRODUCTION

The amygdala and hypothalamus are heterogeneous and highly interconnected divisions of the limbic system. The amygdala plays a crucial role in memory formation, integration of sensory input, and responses to stressful stimuli (reviewed Sah et al., 2003). In particular, the central amygdaloid nucleus (CeA) is the primary output of the amygdala and is involved in the behavioral and physiological responses of fear and anxiety (Davis, 1992). The medial amygdaloid nucleus (MeA) is crucial in appropriate mating behavior and the identification of predators (Lehman and Winans, 1982; Li et al., 2004). The hypothalamus controls pituitary hormone release and much of the body’s physiological function directed by the rest of the brain. The medial preoptic area (mPOA) has been linked to male reproductive behavior and thermoregulation (Liu et al., 1997; Zhang et al., 2011), and the lateral hypothalamus (LH) has been implicated in feeding behavior and some motor aspects of motivation (Lytle and Campbell, 1975). The ventromedial nucleus (VMN) functions in reproductive behavior, feeding, and thermoregulation, and fear responses (Egawa et al., 1991; Cohen and Pfaff, 1992; Flier and Maratos-Flier, 1998).

The developmental trajectory of an animal is particularly vulnerable to external challenges from gestation through adolescence. Endocrine disrupting chemicals (EDCs) administered early in development affect numerous aspects of physiology and behavior (Diamanti-Kandarakis et al., 2009; Crews et al., 2014). Vinclozolin is a fungicide and an anti-androgenic EDC that is an effective antagonist of the androgen receptor (Wong et al., 1995). Vinclozolin exposure of males during gestation causes feminizing effects typical of an anti-androgen including reduced anogenital distance, nipple retention, and cleft phallus with hypospadias (Kelce et al., 1994). The effects of gestational exposure to vinclozolin are known to extend for multiple generations. F3 generation males whose F1 progenitors were exposed to vinclozolin during gestation show increased spermatogenic apoptosis, decreased sperm motility, and premature cancer development (Anway et al., 2006a,b). Exposed F3 males also show behavioral deficits that include aversion by potential mates (Crews et al., 2007), decreased anxiety in aged males (Skinner et al., 2008), and a decreased preference for social novelty (Crews et al., 2012).

The phenotype of an animal is not only a consequence of inheritance but also of an accumulation of the individual’s life experiences. We have previously argued that germline dependent epigenetic modifications, represented in this manuscript by vinclozolin exposure, may influence or interact with context dependent epigenetic modifications (i.e., life experience; Crews, 2011). The current research aims to challenge the phenotype inherited from ancestral vinclozolin exposure with a detrimental life experience. Stress has been well documented to elicit physiological, behavioral, and neurological effects (Padovan and Guimarães, 2000; Isgor et al., 2004; McCormick et al., 2010). Chronic restraint stress is a stressor that is commonly employed in which animals are physically prevented from moving or feeding. Further, adolescence is a particularly vulnerable period of development in which stressful experiences can have long lasting effects (Romeo, 2010). We have previously demonstrated that CRS applied during adolescence reduces anxiety behaviors in adult males (Crews et al., 2012; Gillette et al., 2014).

In this report we mimic a scenario that is more realistic than analyzing a germline and context dependent epigenetic modification in isolation. Vinclozolin exposure three generations previously and chronic restraint stress applied during adolescence were analyzed separately and in combination to determine how the developmental trajectory of the brain was altered. We focus on neural gene expression by qPCR. We find that vinclozolin and CRS primarily affect distinct functional networks of genes and combine to affect expression of methylation machinery in the CeA and thermal regulation in the mPOA. The behavior and physiology of the animals used for these experiments has been previously described (Crews et al., 2012) and we discuss the relevance of the current data both in the context of altered behavior, physiology, and the neural networks in which gene expression is altered.

## MATERIALS AND METHODS

### ANIMALS AND TREATMENT AT F0

Forty eight male Sprague–Dawley rats at ∼120 days of age were used for analysis. All animals were three generations (F3) removed from EDC exposure. F0 gestating dams were injected with either Vinclozolin (100 mg/kg) or DMSO during gestational days 8–14. All breeding was performed within each lineage to yield an F3 generation of pups. No sibling or cousin breeding was allowed. Thus, there were two lineages of animals; one descended from the Vinclozolin and the other from the DMSO treated progenitors. Pups were weaned at postnatal day (PND) 21 and F3 males were shipped from Washington State University to the University of Texas at Austin. Upon arrival, one control lineage and one vinclozolin lineage animal were housed as a pair. Breeding yielded a 4-day variation in age but pairings were restrained to a difference of 1-day. Two cohorts of animals, separated by 4 months, were used for analysis. Each cohort was equally represented in analysis (*n* = 6 per cohort per group). Cohort was included as a covariant but no differences were found. Pairs were housed in standard polycarbonate rat cages (46 cm × 24 cm × 20.5 cm) on a 14:10 light–dark schedule with *ad libitum* access to tap water and standard rat chow (rodent chow 5ll2 Prolab RMH 1800 diet, Purina). Environmental enrichment, as per IACUC regulations, was provided to all pairs (7 cm diameter PVC pipe). All animals were removed from their housing for bi-weekly handling and weighing. All animal protocols were reviewed and approved prior to use by the Institutional Animals Care and Used Committee at both the University of Texas at Austin and Washington State University.

### CHRONIC RESTRAINT STRESS (CRS) DURING ADOLESCENCE

Half of all pairs were randomly selected for CRS. Both animals in a pair were exposed to CRS for 6 h/day for 21 consecutive days and began at PND 26 and ended PND 46. Pairs were taken together to a separate room 1 h after lights out (9:30 AM) and remained in dark through the duration of restraint. Restraint was applied by gently coaxing each animal into a wire-mesh enclosure (25.4 cm^2^) that prevented turning and limited limb mobility. Animals were not allowed access to food or water during restraint stress. Binder clips were used to adjust the size of the enclosure as the animals grew. The health of the animals was tracked and considerable efforts were made to ensure that there was no unnecessary pain or discomfort beyond the confines of our approved protocols. Upon completion of the stress paradigm, animal handling and weighing continued until sacrifice at PND 120 (weight data previously published: Crews et al., 2012). All animals were subjected to a behavioral battery 24-h before sacrifice for which data has already been published (Crews et al., 2012).

### EXPERIMENTAL DESIGN

**FIGURE 1:**
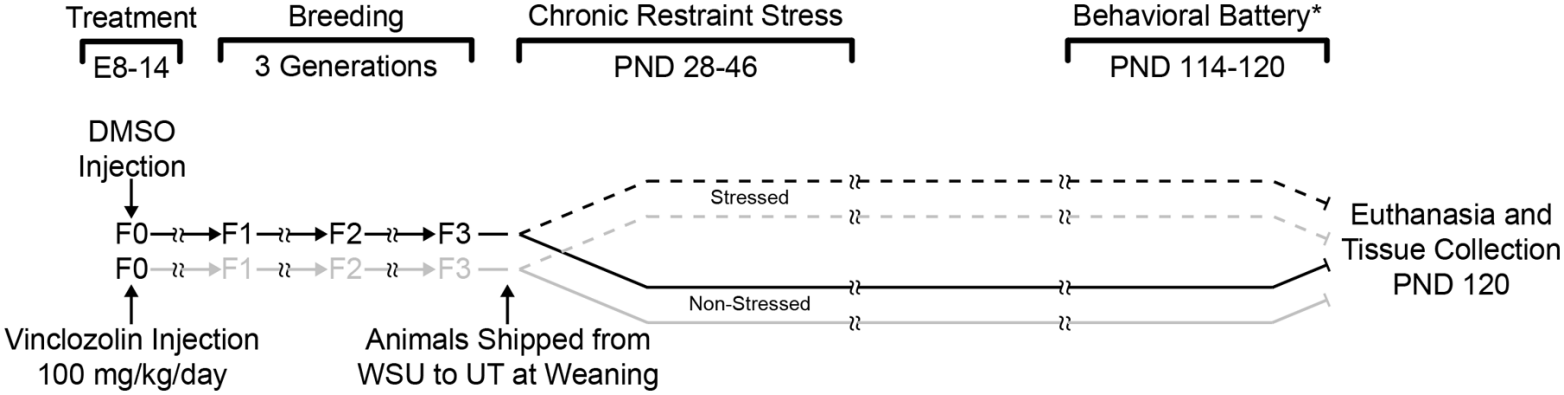
**Experimental design and timeline.** F0 pregnant dams were injected daily with either DMSO or vinclozolin from E8 to E14. Pups were then breed within lineage until the F3 generation. F3 pups were shipped to the University of Texas after weaning. One control lineage (DMSO) and one vinclozolin lineage male was pair housed and half of the pairs were chosen for CRS (6 h/day from PND 28–46). Animals were euthanized and brain tissue was collected at PND 120. (*) a behavioral battery was performed on these animals between PND 114–120; these data have previously been published (Crews et al., 2012).

Two experimental manipulations, ancestral vinclozolin exposure, and CRS, yielded a 2 × 2 design resulting in four groups; Control-Non-Stress (C-NS), Control-Stress (C-S), Vinclozolin-Non-Stress (V-NS), and Vinclozolin-Stress (V-S). See **Figure 1** for a complete timeline of treatment, stress, and resultant groups.

### TISSUE COLLECTION

To insure integrity of the RNA, animals were sacrificed via decapitation and the brain removed within 3-min. Brains were chilled on ice for 5-min and sectioned at 2 mm intervals. Five brain areas were extracted for analysis via micropunch (1 mm from Stoelting); mPOA, LH, VMN, medial amygdala (MeA), and central amygdala (CeA). Punches were stored in eppendorf tubes, frozen on dry ice, and stored at -80°C until RNA extraction.

### RNA EXTRACTION AND qPCR

RNA was isolated and quantified as previously described (Walker et al., 2013; Gillette et al., 2014). Briefly, RNA was isolated from brain punches via silica-membrane spin columns (Allprep RNA/DNA mini kit, Qiagen) and suspended in RNase free water. RNA quantity was established via spectrophotometry (NanoDrop 1000) and quality via ribosomal RNA *28s* to *18s* ratio (RNA 6000 Nano Kit on a 2100 Bioanalyzer, Agilent). RNA samples with an RNA integrity number of less than seven were not used. RNA was diluted and 200 ng was converted to cDNA (High Capacity cDNA reverse transcription kit, Applied Biosystems). PCR was performed with custom designed Taqman Low Density Arrays (TLDA, Applied Biosystems) on a ViiA 7 real-time PCR system (Applied Biosystems).

Two different TLDA cards were used for analysis. Although there was overlap in many genes, the first targeted genes in the amygdala (MeA and CeA) and the second targeted genes in the hypothalamus (mPOA, LH, and VMH). Each TLDA measured the RNA expression of 48 genes targeted in the categories of epigenetic modification, stress signaling, steroid hormone enzymes, and receptors, neuronal communication, peptides, and receptors, growth factors, and transcription factors. Gene names, abbreviations, and their functional groups are shown in **Table 1**. Cycle threshold (Ct) was recorded for each sample and mRNA target during the linear phase of PCR. Ct was first normalized (Δ-Ct) to the geometric mean of each individual sample’s expression of rRNA *18s* and cell cycle gene *Gapdh* and was finally normalized (ΔΔ-Ct) to the median expressing animal of the C-NS group for comparison purposes.

**Table 1:** Gene abbreviations and names.

Category/Gene
**Control genes**		**Glutamatergic**
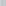 *18s*	Ribosomal subunit 18s	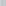 *Gria1*	Glutamate receptor 1
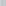 *Gapdh*	Glyceraldehyde-3-phosphate dehydrogenase	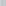 *Gria2*	Glutamate receptor 2
**Epigenetic modification**		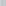 *Grik2*	NMDA (glutamate) receptor ionotropic kainate 2
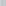 *Dnmt1*	DNA methyltransferase 1	□*Grin1*	NMDA (glutamate) receptor 1
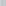 *Dnmt3a*	DNA methyltransferase 3a	□*Grin2a*	NMDA (glutamate) receptor 2a
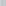 *Dnmt3b*	DNA methyltransferase 3b	□*Grin2b*	NMDA (glutamate) receptor 2b
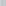 *Dnmt3I*	DNA (cytosine-5-)-methyltransferase 3-like	□*Grin2c*	NMDA (glutamate) receptor 2c
*■Hdac1*	Histone deacetylase 1	□*Grin2d*	NMDA (glutamate) receptor 1
*■Mbd2*	Methyl-binding domain protein 2	**Peptides and receptors**
**Stress signaling**		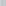 *Avp*	Arginine vasopressin
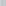 *Crh*	Corticotropin releasing hormone	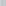 *Avpr1a*	Arginine vasopressin receptor 1A
*■Crhr1*	Corticotropin releasing hormone receptor 1	□*Kiss1*	Kisspeptin
*■Gmeb2*	Glucocorticoid modulatory element binding protein 2	□*Kiss1r*	Kisspeptin receptor (GPR54)
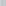 *Nr3c1*	Glucocorticoid receptor	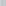 *Lepr*	Leptin receptor
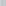 *Pomc*	Proopiomelanocortin	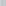 *Mc3r*	Melanocortin 3 receptor
**Steroid-hormone enzymes**		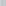 *Mc4r*	Melanocortin 4 receptor
□*Cyp19a1*	Aromatase	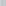 *Mc5r*	Melanocortin 5 receptor
*■Hsd11b2*	Corticosteroid 11-beta dehydrogenase isozyme 2	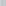 Oxt	Oxytocin prepropeptide
□*Srd5a1*	5-alpha reductase	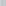 *Oxtr*	Oxytocin receptor
**Sterod-hormone receptors**		□*Tac2*	Neurokinin B (tachykinin 3)
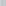 *Ar*	Androgen receptor	**Growth factors**
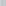 *Esr1*	Estrogen receptor alpha	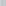 *Bdnf*	Brain derived neurotrophic factor
*■Esr2*	Estrogen receptor beta	*■Ctgf*	Connective tissue growth factor
*■Gnrhr*	Gonadotropin releasing hormone receptor	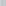 *Igf1*	Insulin-like growth factor 1
□*Gper*	G protein-coupled receptor 30 (GPR30)	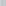 *Igf1r*	Insulin-like growth factor 1 receptor
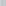 *Pgr*	Progesterone receptor	*■Igfbp2*	Insulin-like growth factor binding protein 2
**Dopaminergic**		*■Igfbp5*	Insulin-like growth factor binding protein 5
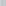 *Comt*	Catechol-*O*-methyltransferase	*■Negr1*	Neuronal growth regulator 1
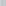 *Drd2*	Dopamine receptor D2	*■Ptgds*	Prostaglandin D2 synthase
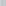 *Drd4*	Dopamine receptor D4	*■S100a4*	s100 calcium binding protein A4
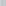 *Th*	Tyrosine hydroxylase	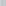 *Tgfa*	Transforming growth factor alpha
**Serotinergic**		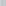 *Tgfb1*	Transforming growth factor beta 1
□*Slc6a4*	Serotonin transporter (family 6, member 4)	**Transcription factors**
**GABAergic**		*■Nfkb1*	Nuclear factor NF-kappa-B
□*Gad1*	Glutamate decarboxylase 1	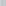 *Nrf1*	Nuclear respiratory factor 1
□*Gad2*	Glutamate decarboxylase 2	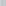 *Per2*	Period circadian clock 2

*■Amygdala Only; 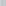
Both Areas; □Hypothalamus Only*.

*Two Taqman Low Density Arrays (TLDA cards, each measuring 48 targeted genes, were used to determine gene expression via qPCR. One card focused on hypothalamic nuclei and the other on amygdaloid nuclei. Gene abbreviations and their full name are shown and are organized by the functional category to which they belong. Genes with a grey box were measured for all nuclei discussed. Genes with a box colored black were measured only in amygdaloid nuclei and genes with a white box were measured only in the hypothalamic nuclei*.

### STATISTICAL ANALYSIS

Statistical analysis was performed within brain area and within gene product. An analysis of variance (ANOVA) contingency table (2 × 2) was used to examine the main effect of ancestral exposure (vinclozolin versus control), CRS (Stress versus Non-Stress), or the relative contributions of lineage and CRS (Interaction). Normality and homogeneity of variance were determined with a Shapiro–Wilk and Levene’s test, respectively. If the data were determined to be non-normal or have heterogeneous variance a Kruskal–Wallis one-way analysis of variance was used to determine effects of ancestral exposure (vinclozolin versus control lineage) or of CRS (Stress versus Non-Stress). *Post hoc* pair-wise comparisons were performed using a Mann–Whitney *U* test.

Finally, in addition to Fisher’s ANOVA, we utilize alternative analytic methods introduced by Hogben. The ANOVA was intended as a formalization of Mendelism and hence is based on the rare phenomenon of traits having a binary nature. Such attempts to deconstruct phenotypic variability using a linear model have consistently proved to have little predictive value (accounting usually for about 5% of the trait). G × E studies as well as more advanced genome-wide association methods are now widely recognized as a failure for understanding complex phenotypes such as disease (Crews and Gore, 2012; Kuzawa, 2012; Rockman, 2012). Hogben (1932), a contemporary of Fisher, proposed “Differences can be described as determined predominantly by heredity or predominantly by environmental agencies if, and only if, the conditions of development are specified.” Hogben’s analysis of the sources of variability extended beyond genetic and environmental sources to a distinct *third class of variability*, which he argued ‘arises from the combination of a *particular* hereditary constitution with a *particular* kind of environment.’ This *class* was essentially developmental in nature. The most appropriate term for this type of third class of variability is *synchronicity*, which describes the coincidence of two factors that are not causally connected (Crews et al., 2014; Gillette et al., 2014). In this instance the control-lineage, Non-Stress group versus the vinclozolin-lineage Stress group. The aim of synchronicity is to delineate an ANOVA interaction from the combination of two acausal phenomena (ancestral vinclozolin exposure and CRS during adolescence).

Indeed, our data indicate that the interaction term from ANOVA and synchronicity fundamentally yield different results and are not synonymous. If warranted, the synchronicity comparison can better help the reader discern the source of a statistical effect and elucidate previously hidden ones. Of the 11 genes that showed either an ANOVA interaction effect or synchronicity, only one gene (*Mc5r* in the mPOA) showed both. This indicates that an ANOVA interaction is not the result of synchronicity but is instead a separate phenomenon.

*Post hoc* comparisons were only performed against the control C-NS group and only if the group to be compared showed at least a 50% increase or decrease of expression. This strict cutoff resulted in far fewer comparisons than all possible combinations and restricts effects to those with biological significance and robust changes in expression. In the cases that multiple comparisons were performed within a gene, a Benjamini–Hochberg false discovery rate correction was applied and *p*-values are reported as such. Outliers were removed using Grubb’s test for outlier and consisted of no more than two individuals per group.

## RESULTS

### CENTRAL AMYGDALOID NUCLEUS (CeA)

**FIGURE 2:**
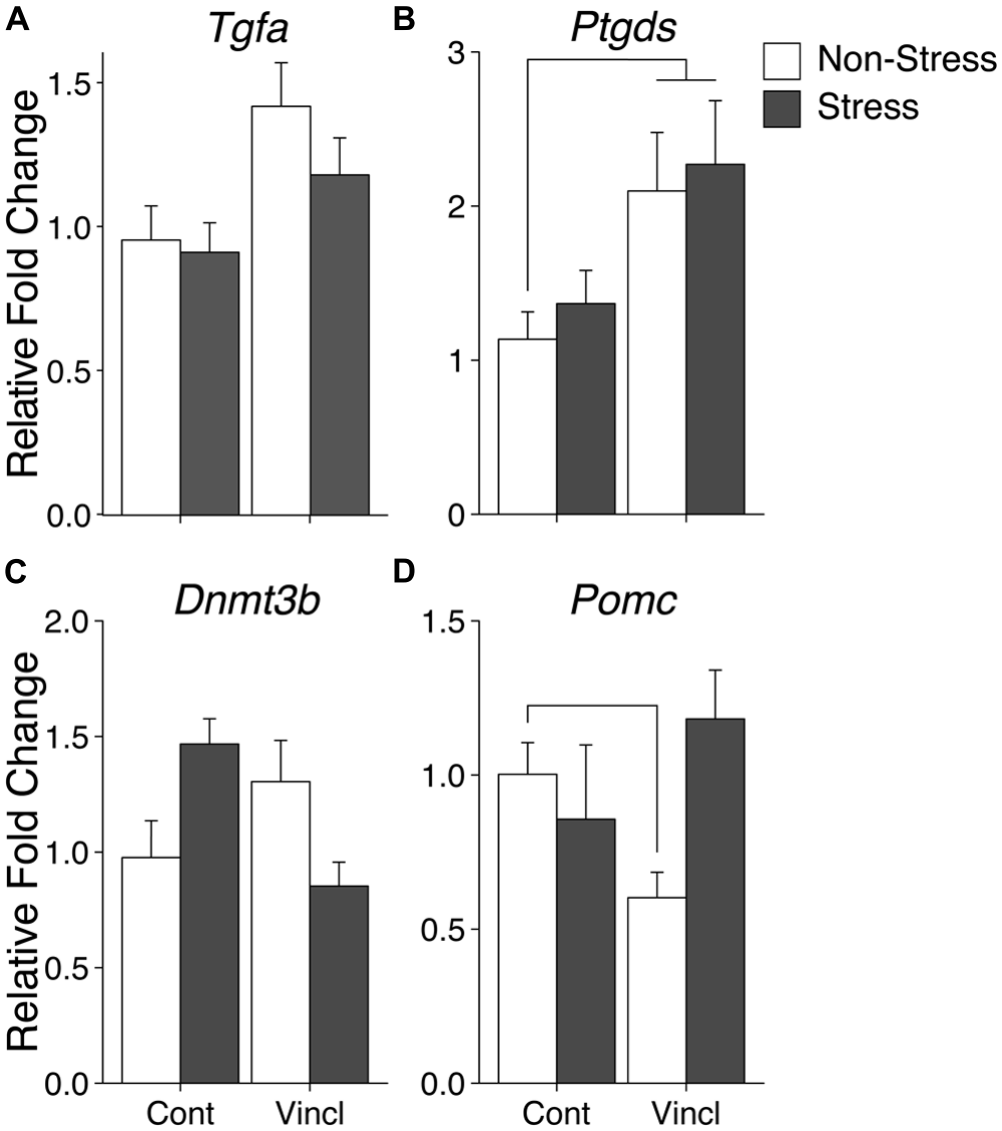
**Gene expression in the central amygdala.** Relative gene expression of Growth factors *Tgfa* and *Ptgds* **(A,B)** show effects of vinclozolin treatment, both up-regulated by ancestral vinclozolin treatment. **(C)** Epigenetic modification enzyme *Dnmt3b* shows an interaction with C-S and V-NS up-regulated and V-S near baseline. **(D)** Stress signaling propeptide *Pomc* shows a significant interaction with C-S and V-NS animals slightly down-regulated. Lines connecting group bars signify a significant *post hoc* pair-wise test.

Ancestral vinclozolin exposure showed effects in an epigenetic modification gene and two growth factors. The methyl binding protein *Mbd2* is thought to be involved the catalyzation of active demethylation and associates with a number of other epigenetic modification proteins. *Mbd2* expression was increased due to ancestral vinclozolin exposure (*H* = 4.08, *p* = 0.043). The neural proliferation factors *Tgfa* and *Ptgds*, which also act as neuromodulators, were affected by vinclozolin exposure and both showed increased expression (*H* = 6.38, *p* = 0.012 and *H* = 6.62, *p* = 0.01, respectively, **Figures 2A,B**). *Post hoc* analysis revealed that expression of *Ptgds* in V-NS males was significantly higher than C-NS males [*W*(21) = 29, *p* = 0.03]. Stress did not significantly affect any of the measured genes in the CeA.

A significant interaction was seen in the epigenetic modification factor *Dnmt3b*, which catalyzes the *de novo* addition of methyl groups to CpG dinucleotides [*F*(1,38) = 10.34, *p* = 0.003]. This interaction is the result of increased *Dnmt3b* expression in C-S and V-NS animals but not V-S animals (**Figure 2C**). C-S animals showed a 49% increase in expression of *Dnmt3b* but did not meet the threshold for statistical comparison. The stress signaling pro-peptide *Pomc* was found to show an interaction of stress and lineage [*F*(1,37) = 6.19, *p* = 0.018, **Figure 2D**] and was due to a significant decrease of expression in V-NS animals and a non-significant increase V-S animals as identified by *post hoc*-analysis [*W*(20) = 100, *p* = 0.01]. *Post hoc* comparison of C-NS versus V-S (synchronicity) indicates increased expression of *Ptgds* [*W*(21) = 30, *p* = 0.03]. A summary of the data and significant effects identified in the CeA are listed in **Table 2A**.

**Table 2:** Amygdala data summary.

Gene	Treatment	Stress	*N*	Mean ± SEM	Effects/synch
**(A) CeA**
*Dnmt3b*	Control	Non-Stress	10	0.98 ± 0.16	Interaction
		Stress	10	1.47 ± 0.11	
	Vinclozolin	Non-Stress	12	1.30 ± 0.18	
		Stress	10	0.85 ± 0.10	
*Mbd2*	Control	Non-Stress	11	1.13 ± 0.11	Effect of lineage
		Stress	11	1.32 ± 0.21	
	Vinclozolin	Non-Stress	11	1.33 ± 0.12	
		Stress	12	1.42 ± 0.10	
*Pomc*	Control	Non-Stress	11	1.00 ± 0.10^2^	Interaction
		Stress	8	0.86 ± 0.24	
	Vinclozolin	Non-Stress	11	0.60 ± 0.08^2^	
		Stress	11	1.18 ± 0.16	
*Ptgds*	Control	Non-Stress	11	1.14 ± 0.18^2^	Effect of lineage/synchronicity
		Stress	11	1.37 ± 0.22^2^	
	Vinclozolin	Non-Stress	12	2.10 ± 0.38	
		Stress	12	2.27 ± 0.41	
*Tgfa*	Control	Non-Stress	12	0.96 ± 0.12	Effect of lineage
		Stress	9	0.91 ± 0.10	
	Vinclozolin	Non-Stress	12	1.42 ± 0.15	
		Stress	11	1.18 ± 0.13	
**(B) MeA**
*Ctgf*	Control	Non-Stress	10	1.07 ± 0.17^1^	Effect of stress/synchronicity
		Stress	10	1.68 ± 0.22^1^	
	Vinclozolin	Non-Stress	10	1.13 ± 0.15	
		Stress	11	1.89 ± 0.29	
*Per2*	Control	Non-Stress	10	1.16 ± 0.17	Effect of lineage
		Stress	10	1.41 ± 0.20	
	Vinclozolin	Non-Stress	10	0.93 ± 0.16	
		Stress	10	0.96 ± 0.10	
*Ptgds*	Control	Non-Stress	9	1.06 ± 0.18	Effect of lineage
		Stress	10	1.18 ± 0.26	
	Vinclozolin	Non-Stress	11	1.70 ± 0.30	
		Stress	11	2.17 ± 0.66	
*Tgfa*	Control	Non-Stress	8	1.15 ± 0.19^1,2^	Effect of stress/synchronicity
		Stress	9	2.07 ± 0.33^1^	
	Vinclozolin	Non-Stress	9	1.62 ± 0.28^2^	
		Stress	10	1.83 ± 0.24

*The mean, SE of the mean, group N, main effects or interactions, and *post hoc* effects are summarized for the **(A)** central amygdaloid nucleus and **(B)** medial amygdaloid nucleus. All *post hoc* comparisons are relative to the control Non-Stress group (C-NS). ^1^Indicates a change of expression in C-S animals and ^2^in V-NS animals. Main effects, interactions, and effects of synchronicity (C-NS vs V-S) are listed within the appropriate gene*.

### MEDIAL AMYGDALOID NUCLEUS (MeA)

**FIGURE 3:**
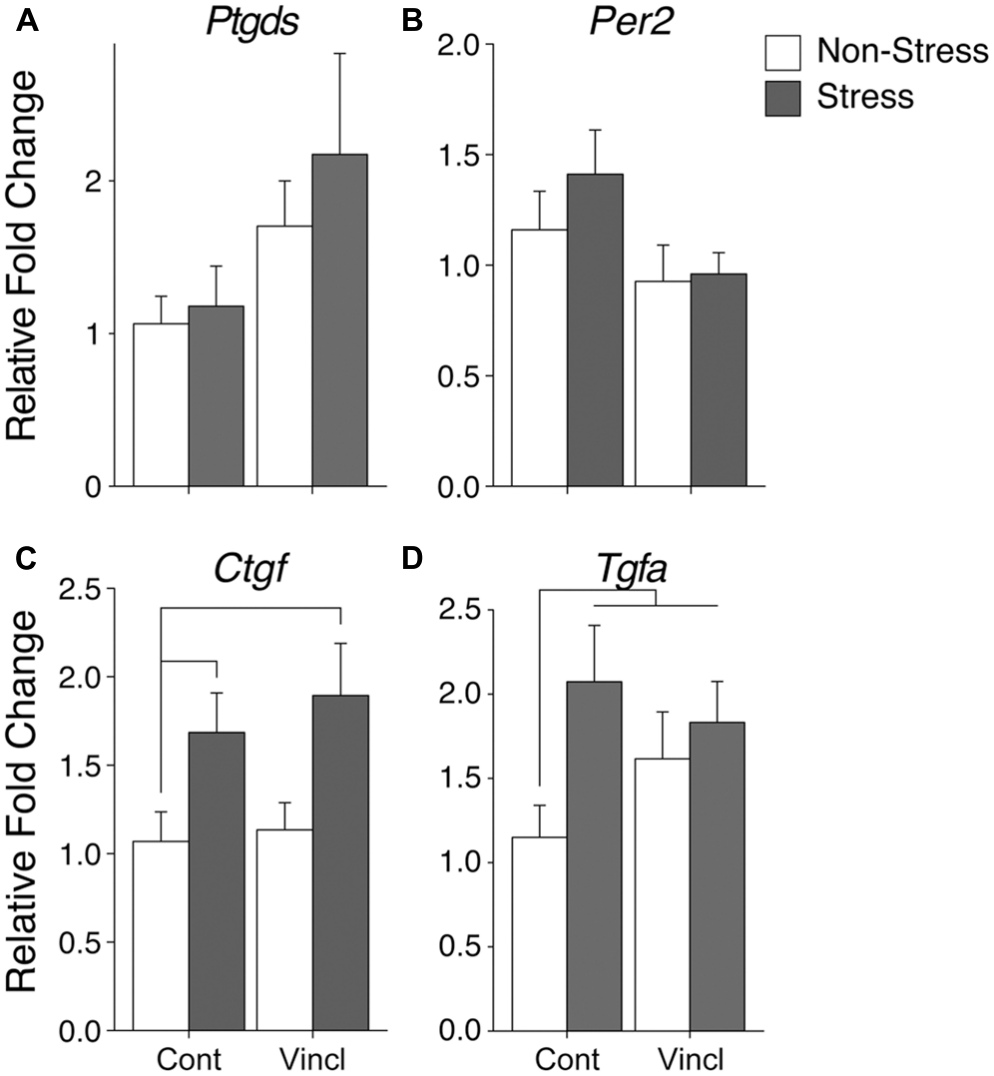
**Gene expression in the medial amygdala**. Relative gene expression of **(A)** Growth factor and neuromodulator *Ptgds* shows a significant effect of ancestral vinclozolin exposure. **(B)** Circadian rhythm gene *Per2* was significantly down regulated by ancestral vinclozolin exposure. **(C,D)** Growth factors *Ctgf* and *Tgfa* show effects of CRS, both up-regulated by CRS during adolescence. *Post hoc Ctgf* comparisons of C-S and V-S were significantly increased compared to C-NS. All three treatment groups (C-S, V-NS, and V-S) were found to be significantly increased in *post hoc* analysis of *Tgfa*. Lines connecting group bars signify a significant *post hoc* pair-wise test.

Ancestral vinclozolin exposure was found to affect two genes in the MeA. Growth gene and neural modulator *Ptgds* was increased (*H* = 5.17, *p* = 0.023, **Figure 3A**). *Per2,* which contains a glucocorticoid response element and modulates glucocorticoid-mediate gene expression, was decreased due to ancestral exposure to vinclozolin (*H* = 4.01, *p* = 0.045, **Figure 3B**). The MeA showed neural proliferation genes were particularly affected by stress. CRS increased the expression of two growth factors in the MeA, *Ctgf* and *Tgfa* (*H* = 8.38, *p* = 0.004 and *H* = 4.58, *p* = 0.032, respectively, **Figures 3C,D**). *Post hoc* analysis of *Ctgf* revealed that C-S animals had a substantial increase in expression of *Ctgf* [*W*(18) = 23, *p* = 0.04, **Figure 3C**]. *Post hoc* analysis was similar for *Tgfa* in C-S animals [*W*(13) = 3, *p* < 0.01, **Figure 3D**]. A significant increase in V-NS animals was also identified [*W*(15) = 11, *p* = 0.02].

No significant interactions were identified in the MeA. *Post hoc* comparisons of synchronicity revealed increased expression of two growth factors, Ctgf, and Tgfa [*W*(20) = 20, *p* = 0.04 and *W*(15) = 11, *p* = 0.02, respectively, **Figure 5B**]. A summary of the data and significant effects identified in the MeA are listed in **Table 2B**.

### MEDIAL PREOPTIC AREA (mPOA)

**FIGURE 4:**
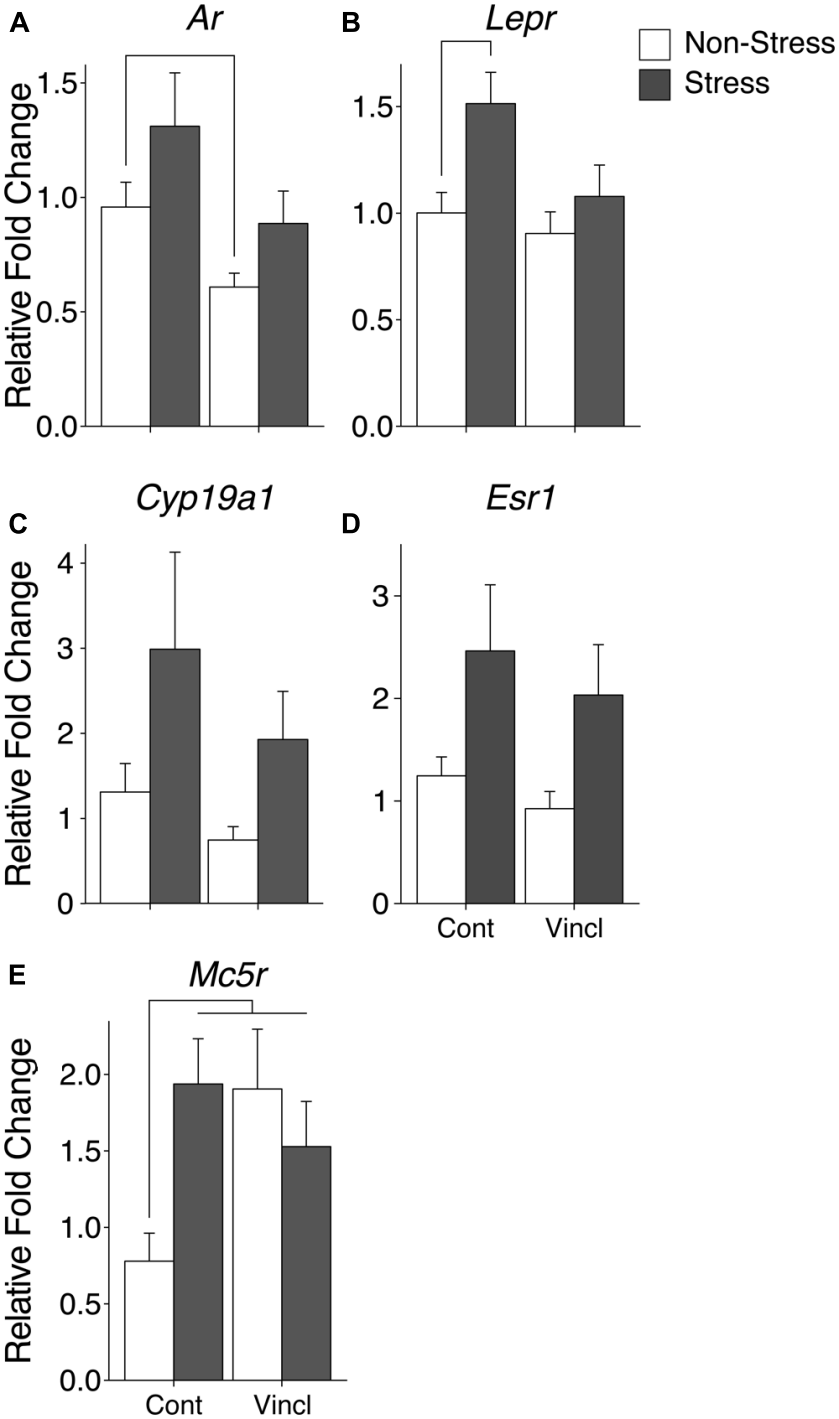
**Gene expression in the medial preoptic area (mPOA).** Relative gene expression of **(A)** Steroid hormone receptor *Ar*, which shows a decrease of expression due to ancestral exposure. **(B)** Peptide receptor *Lepr* shows an effect of vinclozolin treatment, an effect driven by the large increase in expression in C-S animals. **(C,D)** Steroid synthesis enzyme *Cyp19a1* (aromatase) and steroid hormone receptor *Esr1* are both up-regulated by CRS. **(E)** Peptide receptor *Mc5r* shows an effect of vinclozolin exposure. Expression in C-S animals is also increased. Lines connecting group bars signify a significant *post hoc* pair-wise test.

The mPOA was particularly vulnerable to both CRS and vinclozolin exposure compared to the other hypothalamic nuclei analyzed. Both vinclozolin exposure and CRS affected steroid hormone genes and metabolic related genes in the mPOA. Vinclozolin exposure decreased the expression of *Ar* (*H* = 6.14, *p* = 0.013) an effect driven largely by the substantial decrease of expression in V-NS animals [*W*(20) = 96, *p* = 0.02, **Figure 4A**]. *Lepr* expression appears to be decreased due to vinclozolin exposure [*F*(1,37) = 4.73, *p* = 0.036] but *post hoc* analysis reveals that this effect is driven by the increase of expression in C-S animals [*W*(18) = 18, *p* = 0.02, **Figure 4B**]. *Post hoc* analysis revealed that the *Igf1* growth factor ligand was revealed to have increased expression in V-NS animals [*W*(19) = 21, *p* = 0.04]. CRS increased the expression of aromatase (*Cyp19a1*), which catalyzes the conversion of androgens to estrogens, and the estrogen receptor *Esr1* (*H* = 5.37, *p* = 0.021 and *H* = 5.97, *p* = 0.015, respectively, **Figures 4C,D**). CRS also increased the expression of two peptide receptors, the leptin receptor *Lepr* and melanocortin receptor *Mc4r* [*F*(1,37) = 7.46, *p* = 0.010, **Figure 4B** and *F*(1,38) = 4.13, *p* = 0.049, respectively]. The circadian regulation gene *Per2* was found to be slightly but significantly increased due to CRS (*H* = 5.27, *p* = 0.022).

A significant interaction in *Mc5r* expression resulted from the combination of CRS and vinclozolin exposure [*F*(1,30) = 5.36, *p* = 0.028] that is driven by an increase of expression in C-S and V-NS animals [*W*(13) = 4, *p* < 0.01 and *W*(17) = 16, *p* = 0.02, respectively, **Figure 4E**]. *Post hoc* comparisons of synchronicity showed that *Mc5r* expression was increased in V-S animals [*W*(14) = 13, *p* = 0.02, **Figure 4E**]. A summary of the data and significant effects identified in the mPOA are listed in **Table 3A**.

**Table 3:** Hypothalamus data summary.

Gene	Treatment	Stress	*N*	Mean ± SEM	Effects/synch
**(A) mPOA**
*Ar*	Control	Non-Stress	11	0.96 ± 0.11^2^	Effect of lineage
		Stress	10	1.31 ± 0.23	
	Vinclozolin	Non-Stress	11	0.61 ± 0.06^2^	
		Stress	10	0.89 ± 0.14	
*Cyp19a1*	Control	Non-Stress	11	1.31 ± 0.34^1^	Effect of stress
		Stress	10	2.99 ± 1.14^1^	
	Vinclozolin	Non-Stress	11	0.74 ± 0.16	
		Stress	10	1.93 ± 0.57	
*Esr1*	Control	Non-Stress	11	1.25 ± 0.18	Effect of stress
		Stress	10	2.46 ± 0.65	
	Vinclozolin	Non-Stress	11	0.93 ± 0.17	
		Stress	10	2.03 ± 0.49	
*Igf1*	Control	Non-Stress	10	1.03 ± 0.10^2^	No effect
		Stress	10	1.69 ± 0.31	
	Vinclozolin	Non-Stress	11	1.64 ± 0.21^2^	
		Stress	10	1.31 ± 0.22	
*Lepr*	Control	Non-Stress	10	1.00 ± 0.10	Effect of lineage and stress
		Stress	10	1.51 ± 0.15	
	Vinclozolin	Non-Stress	11	0.90 ± 0.10	
		Stress	10	1.08 ± 0.15	
**(B) VMN**
*Dnmt3b*	Control	Non-Stress	12	1.12 ± 0.15^2^	Effect of lineage/synchronicity
		Stress	8	0.92 ± 0.09	
	Vinclozolin	Non-Stress	11	0.58 ± 0.08^2^	
		Stress	11	0.60 ± 0.13	
*Grin2d*	Control	Non-Stress	11	0.98 ± 0.11^1^	Interaction
		Stress	9	1.56 ± 0.20^1^	
	Vinclozolin	Non-Stress	12	1.33 ± 0.16	
		Stress	12	1.25 ± 0.15	
*Igf1r*	Control	Non-Stress	12	1.03 ± 0.06	Interaction
		Stress	9	1.17 ± 0.07	
	Vinclozolin	Non-Stress	12	1.13 ± 0.04	
		Stress	11	0.97 ± 0.05	
*Mc5r*	Control	Non-Stress	7	0.86 ± 0.11	Synchronicity
		Stress	7	1.06 ± 0.17	
	Vinclozolin	Non-Stress	10	1.33 ± 0.25	
		Stress	11	1.42 ± 0.18	

*The mean, SE of the mean, group *N*, main effects or interactions, and *post hoc* effects are summarized for **(A)** the mPOA and **(B)** ventromedial nucleus. All *post hoc* comparisons are relative to the C-NS group. ^1^indicates a change of expression in C-S animals and ^2^in V-NS animals. Main effects, interactions, and effects of synchronicity (C-NS vs. V-S) are listed within the appropriate gene*.

### LATERAL HYPOTHALAMUS (LH)

There were no main effects of CRS, ancestral vinclozolin exposure, or interactions of gene expression in the LH. Similarly, *post hoc* analysis did not identify any significant pair-wise changes in gene expression.

### VENTROMEDIAL NUCLEUS (VMN)

**FIGURE 5:**
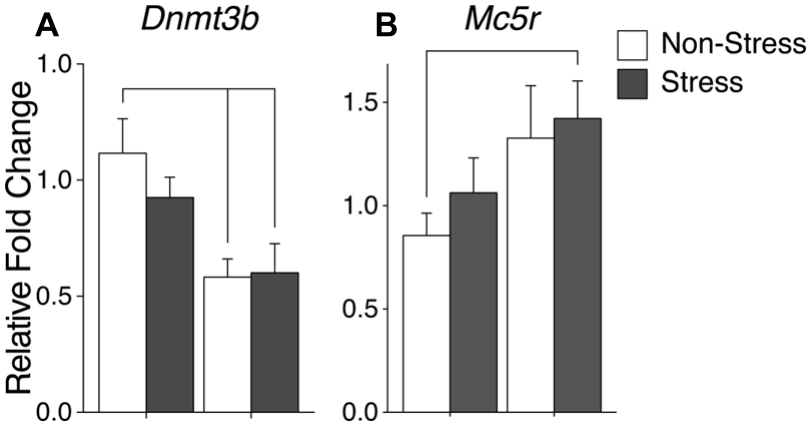
**Gene expression in the ventromedial nucleus (VMN).** Relative gene expression of **(A)** Epigenetic modification enzyme *Dnmt3b* which shows a significant decrease of expression due to ancestral vinclozolin exposure. **(B)** Peptide receptor *Mc5r* shows a significant expression increase in V-S animals. Lines connecting group bars signify a significant *post hoc* pair-wise test.

Ancestral vinclozolin exposure substantially reduced the expression of the epigenetic modifier *Dnmt3b* [*F*(1,38) = 13.86, *p* = 0.001]. *Post hoc* analysis revealed that V-NS animals showed reduced expression of *Dnmt3b* [*W*(21) = 109, *p* = 0.02, **Figure 5A**]. No main effects or *post hoc* effects due to CRS were identified. A significant interaction of CRS and vinclozolin exposure was identified for glutamate receptor subunit *Grin2d* [*F*(1,40) = 4.56, *p* = 0.041] which in large part was driven by a significant increase identified by *post hoc* analysis in C-S animals [*W*(18) = 19, *p* = 0.02].

A significant interaction was evident in growth factor *Igf1r* although changes in individual group expression are minimal [*F*(1,40) = 8.27, *p* = 0.006]. *Post hoc* comparisons of synchronicity showed that V-S animals had reduced *Dnmt3b* expression and [*W*(21) = 101, *p* = 0.03, **Figure 5A**]. Synchronicity was also present in the melanocortin receptor *Mc5r* of V-S animals, which plays an integral role in energy homeostasis and feeding behavior [*W*(16) = 16, *p* = 0.05, **Figure 5B**]. A summary of the data and significant effects identified in the VMN are listed in **Table 3B**.

## DISCUSSION

The amygdala and hypothalamus integrate incoming sensory information, monitor the internal state, and provide appropriate behavioral and physiological response. Insults to the delicate milieu of gene expression of neurons within these areas can ultimately manifest as altered, and sometimes inappropriate, behavior. We have previously shown that both behavior and physiology in adulthood is altered due to vinclozolin exposure three generations previously and CRS experienced during adolescence (Crews et al., 2012). Here we discuss gene expression changes within identified amygdaloid and hypothalamic nuclei derived from the same animals for which the behavior and physiology has been previously reported (Crews et al., 2012).

### GROWTH FACTORS AND THE AMYGDALA

Growth factors play a crucial role in forming and remodeling neurons and their connections in the brain. Neural growth factors alter firing frequency and direct the axonal and dendritic extensions of the neurons to provide additional and appropriate connections to its neighboring and distant targets (McAllister et al., 1999; Singh et al., 2006; Davis-López de Carrizosa et al., 2010). Published behavioral data on the animals currently used for gene expression analysis indicate that ancestral exposure to vinclozolin and CRS during adolescence reduces anxiety behaviors in adulthood. We found that ancestral vinclozolin exposure induces growth factor expression in the MeA and CeA. Three growth factors were affected by ancestral vinclozolin treatment in the amygdala; *Tgfa*, *Ptgds*, and *Ctgf*. A reduction of *Tgfa* has been shown to reduce the vasculature and volume of the CeA which consequently impairs the acquisition and response to stressful memories (Burrows et al., 2000). *Ptgds* synthesizes prostaglandin D2 (Pgd2), which has functions as a neuromodulator and inflammatory response element (Eguchi et al., 1999; Mohri et al., 2003). *Pgd2* is highly expressed in limbic brain regions, including the amygdala (Yamashita et al., 1983), however, its function in the amygdala has not been tested. In correlation with our previously published behavioral findings, our data suggest that *Ptgds*, and therefore *Pgd2*, is involved in the perception and reaction to stressful environments

Following CRS exposure the firing activity of neurons and dendritic arborization in the amygdala is altered (Vyas et al., 2002; Zhang and Rosenkranz, 2012) and plasticity is affected (Cordero et al., 2005). The amygdala is susceptible to environmental input and may permanently be affected by stressful experiences. It is possible there are mechanisms in place to both protect the amygdala and prepare the individual for further stressful events later in life. Our data indicates that the expression of at least two growth factors (*Tgfa* and *Ctgf*) is altered by CRS in adolescence that persist into adulthood. *Ctgf* is a cytokine that is activated in response to traumatic brain injury and has been linked to the neurodegenerative effects of Alzheimer’s (Ueberham et al., 2003; Liu et al., 2014). *Tgfa*, has effects on vasculature and volume growth in the amygdala (Burrows et al., 2000). The current data suggest that these genes are involved in the protective and proliferative effects produced by CRS in adolescence.

### EPIGENETIC MODIFICATION FACTORS

DNA methylation has been most thoroughly considered for its role in cellular differentiation and considered to be a static process. We now know that DNA methylation and demethylation are highly dynamic processes that are necessary for proper cognitive function (Miller and Sweatt, 2007; Sultan et al., 2012). *Mbd2* is a protein cofactor that has been linked to demethylation activity and transcriptional activation *in vivo* and *in vitro* although it does not itself contain catalytic activity (Hendrich and Bird, 1998; Wang et al., 2013). The likely role of *Mbd2* is to recruit demethylation machinery to marked loci in the genome for broad transcriptional changes. *Dnmt3b* catalyzes the addition of methylation to cytosine residues generally suppressing gene expression (Li et al., 1993; Okano et al., 1999). We show that *Mbd2* expression is increased due to ancestral vinclozolin exposure in the CeA. We do not elucidate which specific genes *Mbd2* is associating with or its efficacy. Therefore its specific functional significance cannot be determined. However, its increased expression is indicative of a permissible state of gene expression by its presumed association with promoter demethylation. This seemingly lies in contrast to the inverse-U curve pattern of expression shown by *Dnmt3b* in the CeA. In a dynamic system where gene promoters are being methylated and demethylated, *Mbd2* and *Dnmt3b* are in constant opposition to one another. Taken together, an intriguing possibility of their combined expression arises; C-S animals could be trending toward a transcriptional repressed state, V-S animals toward a transcriptionally permissive state, and V-NS animals to a highly dynamic state This is one of the few instances in which ancestral vinclozolin exposure and CRS affect a similar molecular mechanism. However, the promiscuous and non-specific nature of these factors, which have the ability to ubiquitously alter gene expression, mark methylation machinery a likely point of interaction between a germline- and context-dependent mechanisms.

### STEROID RECEPTORS IN THE mPOA

The mPOA is essential for male copulatory behaviors. Lesions to the mPOA and inhibition of the androgen receptor negatively affect a male rats’ ability to successfully copulate (Liu et al., 1997; Harding and McGinnis, 2004). We find that *Ar* expression in the mPOA is decreased in the vinclozolin lineage. This is an interesting finding considering male rats from an ancestrally exposed vinclozolin lineage are avoided by potential female mates (Crews et al., 2007). Copulatory success due to vinclozolin treatment compared to control animals has not been directly assayed. It is possible that males are providing subtle behavioral cues that females are avoiding or at minimum, perceived as less enticing when provided the opportunity to associate with a healthy unaffected male. This may be a consequence of decreased *Ar* expression. CRS was also found to have effects on steroid hormones in the mPOA. Estrogen receptor α(*Esr1*) and the enzyme that converts testosterone to estrogen (Aromatase – *Cyp19a1*) are up-regulated in the mPOA due to stress. Estrogen signaling in the mPOA plays a crucial role in male mating behavior (Paisley et al., 2012). That both the estrogen receptor and the enzyme that produce estrogen are altered due to CRS in the mPOA may indicate that chronic stress during adolescence affects mating behaviors through adulthood.

### THERMOREGULATORY FACTORS IN THE mPOA

Endocrine disrupting chemicals can influence body weight and produce obesity phenotypes, regardless of their target steroid receptor molecule (Newbold et al., 2005) and can transcend multiple generations after initial exposure (Manikkam et al., 2013). Previous work in our lab suggests that ancestral treatment of vinclozolin results in increased body weight throughout life (Crews et al., 2012). Further, animals exposed to CRS during adolescence gain weight very slowly compared to their non-stressed counterparts but then rebound fairly quickly after the cessation of the CRS paradigm (Crews et al., 2012). The mPOA is involved in the leptin signaling cascade initiated by brown adipose tissue to regulate thermogenesis (Zhang et al., 2011) and brown adipose tissue activity increases preceding *ad libitum* feeding (Blessing et al., 2012). The mPOA is also a target of melanocortin signaling which also modulates thermal regulation and feeding behavior (Hwa et al., 2001; Kishi et al., 2003). We found that CRS increases *Lepr* and *Mc4r* expression in adulthood, however, the increase of *Lepr* due to CRS was not seen in animals exposed to both CRS and ancestral vinclozolin exposure. *Mc5r* expression was increased in all three treatment groups. Taken together, our data suggests that both ancestral vinclozolin exposure and CRS during adolescence can alter thermal regulation circuits independently except in the case of *Lepr* in which vinclozolin alters the phenotype elicited by CRS. It is possible that increased weight due to EDCs is in part due to altered thermal regulation circuitry.

## CONCLUSION

Both germline- and context-dependent epigenetic modifications can influence the physiology and behavior of an animal. Ancestral vinclozolin exposure and CRS are examples of those epigenetic modifications and have both been shown to produce modified phenotypes. The current data provide insight into how these affects may manifest. Germline- and context-dependent modifications have the ability to interact in a number of ways to influence the phenotype; additively, synergistically, or independently. Our data suggest that ancestral vinclozolin exposure and CRS independently influence the phenotype and interact at the level of DNA methylation machinery, a broad and likely point of interaction.

## Conflict of Interest Statement

The authors declare that the research was conducted in the absence of any commercial or financial relationships that could be construed as a potential conflict of interest.
